# Caecal herniation through the foramen of Winslow

**DOI:** 10.1259/bjrcr.20150330

**Published:** 2016-05-18

**Authors:** Gary Tse, Tamas Sollei, Syed Mohammed Ali, Neil Kukreja

**Affiliations:** ^1^ School of Biomedical Sciences, Li Ka Shing Faculty of Medicine, The University of Hong Kong, Hong Kong, China; ^2^ Department of General Surgery, Medway Maritime Hospital, Kent, UK

## Abstract

Internal hernia is the protrusion of an abdominal viscus through the peritoneum or mesentery into a compartment within the abdominal cavity. We present a case of internal herniation through the foramen of Winslow that was identified by CT imaging. It was treated with reduction at laparotomy and subsequent right hemicolectomy.

## Summary

Internal hernia refers to the protrusion of an abdominal viscus through the peritoneum or mesentery into a compartment within the abdominal cavity and represents an uncommon cause of bowel obstruction. Six main types of internal herniation have been described, which are paraduodenal, foramen of Winslow, transmesenteric, paracaecal, intersigmoid and paravesical. Of these, protrusion through the foramen of Winslow is rare and accounts only for approximately 8% of all internal herniae.^1^ Various organs have hitherto been found to herniate through this foramen. Here, we report a case of caecal herniation that was only diagnosed by a CT scan.

## Clinical presentation

A 57-year-old male presented with a 1-day history of sudden onset epigastric and chest pain, associated with nausea, coffee ground vomiting and rectal bleeding. On systemic enquiry of the gastrointestinal system, there was no history of abdominal distension and the patient reported bowel opening the day prior to admission. He had no past history of abdominal surgery but suffered from Type 2 diabetes mellitus and learning difficulties. Clinical examination revealed mild epigastric tenderness in the absence of guarding and rigidity, but was accompanied by reduced bowel sounds, with no evidence of inguinal or femoral herniation.

**Figure 1.**
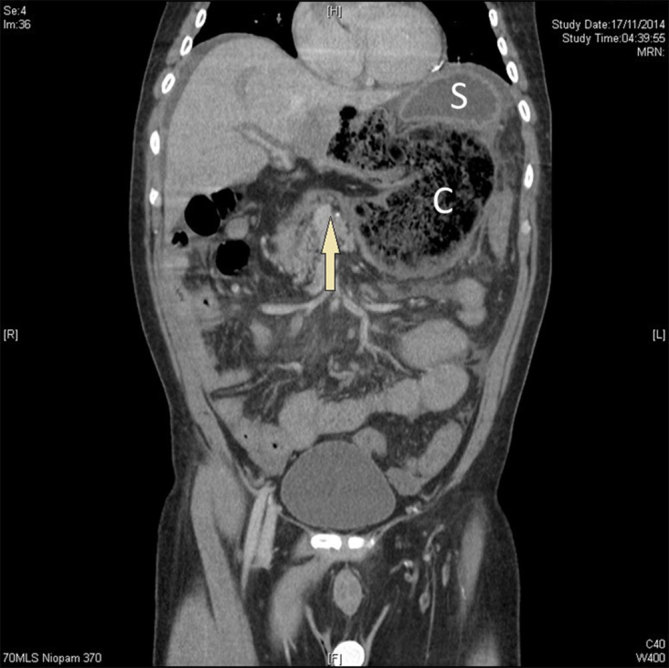
A coronal section of the CT scan showing the caecum (C) herniating through the foramen of Winslow (arrow), lying below the stomach (S).

**Figure 2.**
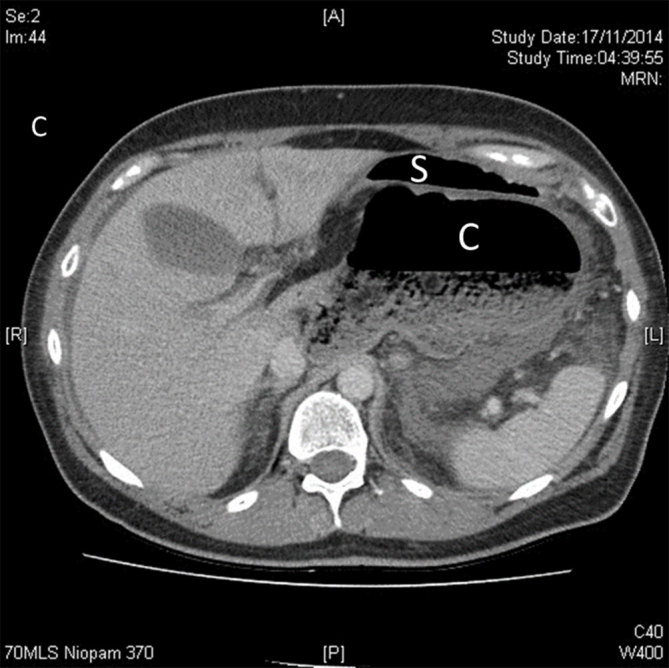
An axial section of the CT scan showing the caecum (C) behind the stomach (S).

On admission, he was apyrexial and results of blood test showed an increased white cell count (16.1, normal range 4–11 × 10^9^ l^–1^), amylase (279, normal range 30–123 U l^–1^) and alanine transaminase (60, normal range 9–55 U l^–1^), as well as deranged renal function tests (creatinine, 151, normal range 36–107 µmol l^–1^; urea, 15.5, normal range 2.5–7.8 mmol l^–1^). Erect chest and abdominal radiography demonstrated gas collection under the hemidiaphragm within a bowel loop but no free air, as well as a distended stomach. The patient was admitted under the medical team with a differential diagnosis of acute coronary syndrome, but his electrocardiogram and troponin were unremarkable. A surgical opinion was therefore requested. An urgent CT scan of the abdomen and pelvis demonstrated large pockets of gas and faeces in the left hypochondrium adjacent to the stomach. This was suggestive of an abnormally lying caecum, secondary to either internal herniation or a volvulus. There was no evidence of free gas in the abdomen. Figure 1 shows a coronal section of the CT scan. Here, the stomach (S) is lying below the left hemidiaphragm with the caecum (C) below it and passing through the foramen of Winslow. An axial view is shown in Figure 2, where the caecum is located behind the stomach.

**Figure 3.**
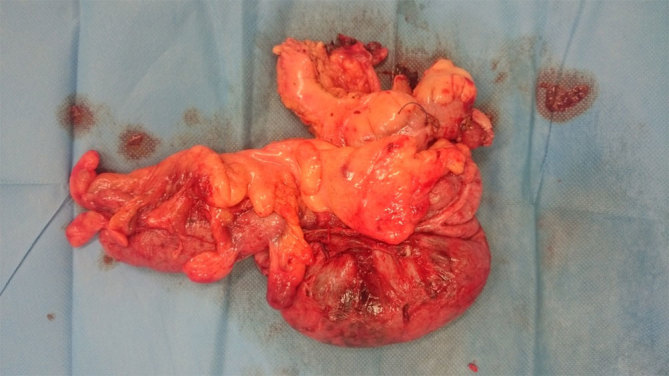
A picture of the resected right colon.

The patient underwent urgent laparotomy that confirmed an internal hernia of the caecum through the foramen of Winslow, lying behind the stomach in the lesser sac causing small bowel obstruction. The caecum showed evidence of ischaemia and necrosis, but there was no abdominal contamination (Figure 3). During surgery, after gaining access *via* the lesser sac, the caecum was decompressed and subsequently reduced through the foramen. A right hemicolectomy was performed with a side-to-side stapled anastomosis. Postoperatively, the patient was transferred to the high dependency unit, went on to have an uneventful recovery and was discharged home a week later.

## Discussion

Internal hernia refers to the protrusion of an abdominal viscus through the peritoneum or mesentery into a compartment within the abdominal cavity.^2^ It is an uncommon cause of small bowel obstruction, accounting for around 5% of all cases.^3^ Timely diagnosis is crucial because internal herniae are associated with a high mortality rate.^3^ Various types have hitherto been described, including paraduodenal, foramen of Winslow, transmesenteric, paracaecal, intersigmoid and paravesical.^4^ Of these, protrusion through the foramen of Winslow represents 8% of internal herniae^1^ and only 0.1% of all abdominal herniae.^5^ This foramen forms the communication between the greater peritoneal cavity and the lesser sac. Its anterior border is the hepatoduodenal ligament and posterior border is the inferior vena cava. Its superior border is the caudate lobe of the liver and inferior border is the start of the duodenum and the hepatic artery. Several organs have been reported to herniate through the foramen of Winslow, including the small bowel, caecum, and ascending and transverse colon.^6^ Involvement of the gallbladder,^7^ small bowel diverticulum^8^ or Meckel’s diverticulum^9^ has rarely been reported. Predisposing factors include an enlarged foramen of Winslow, failed retroperitonealization of the right colon, a long small bowel mesentery and a common intestinal mesentery.^6^ Additional factors are a sudden increase in intra-abdominal pressure^10^ and an atrophic greater omentum.^11^ This disease has a male preponderance, affecting males 2.5 times more frequently than females,^12^ with the greatest incidence between 20 and 60 years of age and a reduced likelihood at extremes of age.^13^

Caecal herniation is a rare event. A caecal volvulus contained in a giant ventral hernia has previously been described in a patient with an extensive history of intra-abdominal surgeries and recurrent ventral hernia.^14^ There was another case involving a giant left-sided Amyand's hernia, where a mobile caecum was entrapped with the appendix within an inguinoscrotal hernia.^15^ Caecal herniation through the foramen of Winslow into the lesser sac has been reported. One case involving not only the caecum but also the proximal ascending colon and terminal ileum has been described.^16^ A different case described herniation of a caecal bascule, where the caecum folded anteriorly and superiorly.^17^ Interestingly, in our case, there was isolated involvement of the caecum in the absence of a bascule.

Accurate diagnosis of internal herniation is challenging. Symptoms can vary greatly, depending on the severity.^2^ It may start with non-specific abdominal pain. If the herniae are spontaneously reducible, intermittent obstructive symptoms can occur. In cases where these are irreducible, acute obstruction ensues and finally strangulation causes peritonism.^18^ In addition, it has been pointed out that unique symptoms are specific for certain types of hernia, correlating with their anatomical variations.^2^ For example, post-prandial pain occurs in paraduodenal herniae.^3^ Haemorrhoids occur in left-sided paraduodenal herniae owing to portosystemic shunting with compression of the inferior mesenteric vein.^19^ Right iliac fossa pain occurs frequently in pericaecal herniae. Transmesenteric herniation often presents as a painful, palpable abdominal mass.^4^ Relevant to our case, foramen of Winslow herniation can cause vomiting owing to gastric compression. It can also cause jaundice if the common bile duct is compressed, which obstructs bile flow.

Different imaging modalities can be used to reach the diagnosis. Radiological signs can vary depending on the organ herniating into the lesser sac. Plain abdominal radiography typically shows anterolateral displacement of the gastric bubble by a mass in the lesser sac.^11^ Evidence of caecal involvement includes absent bowel gas in the right iliac fossa, finding of transverse colon inferior to the distended stomach loop and the presence of an ileal loop on the right side of the abdomen towards the Morison’s pouch.^11^ A CT scan may demonstrate mesenteric fat behind the portal vein, common bile duct and hepatic artery. It may reveal gas or fluid in the lesser sac with a bird’s beak appearance, with the beak pointing towards the foramen of Winslow. If the caecum is involved, it is absent from the normal anatomical position.^20,21^ Narrowing of the portal vein associated with periportal oedema has been described.^22^

Definitive treatment requires prompt surgical correction because of the risk of strangulation of the hernia contents.^23^ Successful surgery has been achieved with a laparoscopic approach,^24^ but open procedures are frequently needed. During laparotomy, reduction of the hernia contents can be achieved by application of gentle pressure.^25^ If this fails, the foramen of Winslow can be enlarged using a Kocher manoeuvre, in which exposure of the retroperitoneal structures is achieved by incising the peritoneum at the right edge of the duodenum and reflecting the duodenum to the left. Resection of the compromised bowel is needed. There is some debate regarding whether the foramen should be closed or not, because attempts at closure may risk damage to the biliary ducts, or hepatic artery and portal vein thrombosis.^23^ Closure has been suggested to be unnecessary as recurrence is unlikely,^26^ and in our case, the omentum was placed in the foramen.

This case study demonstrates variable clinical signs that a patient with an internal hernia can present with and highlights the usefulness of CT imaging in correlation with clinical examination.

## Learning points

1. Patients with internal herniation can present with chest or epigastric pain.
2. CT imaging is important for the assessment of abnormal anatomy.
3. Urgent laparotomy is needed if there are clinical signs of necrosis or diagnosis is uncertain.

## Consent

We confirm that informed consent has been obtained from the patient and we have full permission to publish this case and the images included herein.
